# Anatomical Study of the Tibialis Posterior Tendon's Connections to the Plantar Muscles and Its Relationship With the Severity of Hallux Valgus

**DOI:** 10.1002/jfa2.70074

**Published:** 2025-08-13

**Authors:** Turan Koç, Zeliha Kurtoğlu Olgunus, Fatih Çiçek, Alev Bobuş Örs

**Affiliations:** ^1^ Faculty of Medicine Department of Anatomy Kahramanmaraş Sütçü İmam University Kahramanmaraş Turkey; ^2^ Faculty of Medicine Department of Anatomy Mersin University Mersin Turkey; ^3^ Faculty of Medicine Department of Anatomy Niğde Ömer Halisdemir University Niğde Turkey

**Keywords:** adductor hallucis muscle, flexor hallucis brevis muscle, foot, hallux valgus, tibialis posterior muscle

## Abstract

Changes in tendon morphometry around first‐row bones are linked to the hallux valgus (HV) development. However, there are very limited studies examining the relationship between the connection status of the tibialis posterior (TP) tendon to the adductor hallucis (ADH) and flexor hallucis brevis (FHB) tendons and the development of HV. This study aimed to investigate the association between these tendon connections and the occurrence of HV. The study included 24 formalin‐fixed adult cadavers and amputee feet (10 female, 14 male). The attachment sites and connections between the ADH, FHB, and TP tendons were recorded. Feet were classified into three groups: no connection between the three tendons (Group I), connection between TP and FHB (Group II), and connection between TP, FHB, and ADH (Group III). HV angle values and subgroups (normal, mild, and moderate‐severe) were defined to assess the degree of HV. Feet were grouped based on tendon attachment status, and the distribution of HV subgroups was statistically analyzed. HV angles in Group III were significantly larger than in Groups I and II (= 0.000, *p* = 0.024). While tendon connection was detected in only 20% (1/5) of feet without HV, tendon connection was detected in 64% (7/11) of mild HV and in all feet with moderate‐severe HV. HV occurred in 93.8% (15/16) of feet with tendon connections (vs. 50% without). The study revealed that HV can develop in feet with and without tendon attachments. However, HV is more frequent in cases where the TP tendon is attached to the FHB and ADH tendons. Additionally, moderate‐severe HV increases when TP is attached to ADH.

## 1 Introduction

Hallux valgus (HV) is a progressive deformity that develops in stages. It is one of the most common foot deformities in adults [1]. In the early stage of HV, the big toe (hallux) deviates to lateral and the first metatarsal bone to medial [2, 3, 4]. Progressive subluxation of the first metatarsophalangeal joint (MTPJ) occurs in later stages. The metatarsal head may sublux medially and displace and slide according to the sesamoid. It is suggested that a tilted or unstable tarsometatarsal joint may also encourage this movement. In the third step, the proximal phalanx assumes a valgus position because it is connected to the sesamoids at its base, the deep, transverse ligament (via the plantar plate), and the adductor hallucis tendon (ADH) [5, 6]. Subsequently, the metatarsal head sits on the medial sesamoid and may erode the cartilage and crista. These changes result in the lateral sesamoid appearing to sit in the intermetatarsal space, although it does not move. In the later stage, the bursa covering the medial eminence may thicken due to the pressure effect on a prominent medial prominence, also depending on the shoe worn. The extensor and flexor hallucis longus tendons appear to bend laterally, increasing the valgus displacement and sometimes even acting as dorsiflexors of the proximal phalanx. As the metatarsal head descends from the sesamoid bone, the pronation movement occurs due to the muscular forces passing over it. The cause of HV has been debated for years. It is reported that the underlying factors may be a genetic predisposition [7, 8, 9], restrictive shoe use [10, 11], hypermobility and pes planus [12, 13]. The foot's intrinsic muscles play an important role in its function. Although its anatomy is well‐defined, its functions have not been fully elucidated. However, especially in the orthopedic literature, it is reported that IM is not a subject that is discussed much in terms of the importance of foot pathologies, evaluation, and treatment approaches, and therefore the anatomy, functions, and pathologies of these muscles are ignored [14]. Sizes of intrinsic muscles are small and difficult to assess functionally but are thought to be important in both normal function and common clinical‐pathological processes [14]. Normally, there is a muscle balance in the mediolateral plane of the big toe. The medial tendon of the flexor hallucis brevis (FHB) and abductor hallucis (ABH) is in balance with the lateral tendon of the FHB and ADH [15]. However, it has been reported that this balance may be disrupted if there is tendon transfer or slippage to one side of the big toe in the medial‐lateral plane. USG and electromyography studies have reported that some IMs in HV cases differ from normal cases in terms of size, quality, and function [15, 16, 17, 18]. It is unclear whether HV occurs due to muscle weakness due to aging or whether muscle weakness develops due to deformity [18]. Moreover, it is stated that muscle imbalance in the ABH and ADH muscles is evident in HV and may be the cause or consequence of joint deformity [15]. Weakness or muscle hypertrophy is considered an example of adaptation in the muscle balances in the foot [19]. Our previous study presented that changes in tendon dimensions were evident in severe HV cases but did not differ in mild HV cases. We also suggested that the changes were not due to a factor limited to IMs [20]. Limited studies are found to determine whether there are various connections related to the ADH or whether this organization is associated with deformity. Interestingly, in 1994, it was reported that there may be a connection between the tibialis posterior (TP) and ADH tendons in the presence of HV [21]. However, since that year, we have not encountered any other studies attributing a similar relationship to TP and ADH or TP, ADH and FHB.

This study aimed to evaluate the possible tendinous connections between ADH, TP, and FHB in the presence of HV and their relationship to HV severity.

## 2 Material and Methods

Our results evaluating the relationship of HV with the position and morphometry of tendons and nerves in the region have been published previously [20, 22]. In this study, dissection was performed on 24 formalin fixed cadaveric and amputated feet (10 female, 14 male; age 67.17 ± 13.10 years).

### 2.1 Parameters and Measurements

**FIGURE 1:**
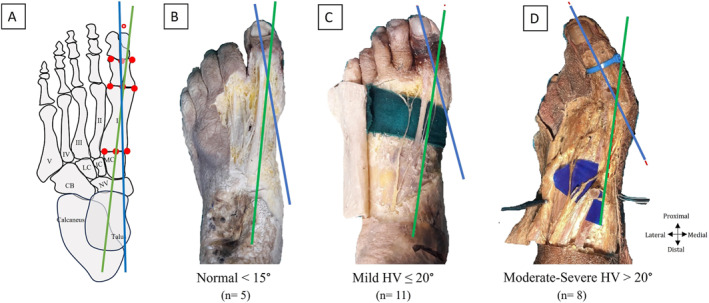
The classification of the feet according to the HV severity. (A) Determination of HV angle (B) Normal, (C) Mild HV, and (D) Moderate‐Severe HV. Red circle: angle between axis of proximal phalanx and I metatarsal bone, blue line: axis of proximal phalanx, CB: cuboid bone, green line: axis of I metatarsal bone, HV: hallux valgus, IC: intermediate cuneiform bone, I‐V: I‐V metatarsal bones, LC: lateral cuneiform bone, MC: medial cuneiform bone, NV: navicular bone.

**FIGURE 2:**
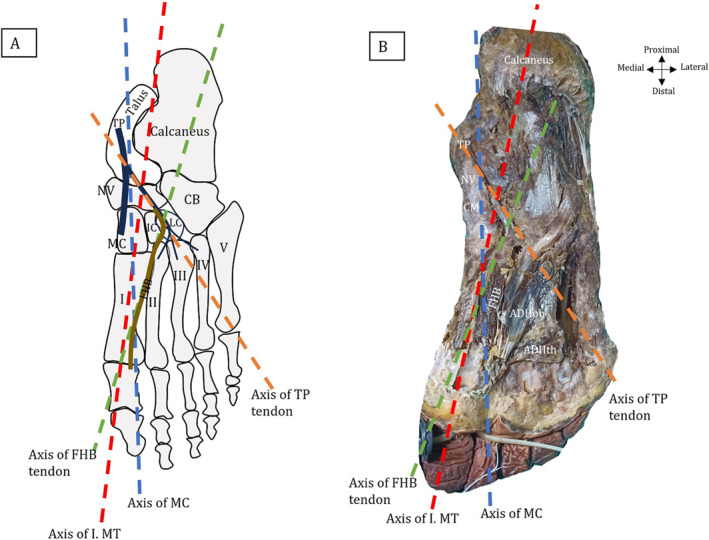
Measurements of angle parameters. (A) Schematic drawings of angle parameters (B) Angle parameters are shown in cadaver. ADHoh: oblique head of adductor pollicis muscle, ADHth: transverse head of adductor pollicis muscle, CB: cuboid bone, FHB: flexor hallucis brevis muscle, IC: intermediate cuneiform bone, I‐V: 1‐5 metatarsal bones, LC: lateral cuneiform bone, MC: medial cuneiform bone, MT: metatarsal bone, NV: navicular bone, TP: tibialis posterior muscle.

- For measurement of the HV angle (HVA): The foot was positioned in a neutral stance. The proximal (closer to the ankle) and the distal ends (closer to the toes) of the first metatarsal bone were identified, and the midpoints of these ends were marked. Similarly, the midpoints of the proximal and distal ends of the proximal phalanx (big toe) were identified and marked (Figure 1A). Flexible rope was used to connect the midpoints of the proximal and distal ends of the first metatarsal bone. This line represents the midline of the first metatarsal. Similarly, the same method was applied to the proximal phalanx to establish its midline. The goniometer's center point was placed at the intersection of the midlines of the first metatarsal bone and proximal phalanx. One arm of the goniometer was aligned to the midline of the first metatarsal bone, and the other was aligned with the proximal phalanx's midline. The angle was recorded directly from the goniometer scale. All values and subgroups for HV level (non‐HV or normal < 15°, mild HV ≤ 20°, and severe to moderate HV > 20°) have already been defined and published previously [20].
- The connections between the TP, oblique head of ADH (ADHoh), and FHB muscle tendons were evaluated, and grouping was performed according to connection status. Muscle connection angulations and relations were evaluated for each group. Muscle connection data related to this section were also shown in the tendon classification section in the previous study [23].
- The following parameters were recorded to reveal whether muscle connection angulations are related to HVA (Figure 2):
- Angle was defined between I. metatarsal bone axis and medial cuneiform bone axis.
- Angle was defined between I. metatarsal bone axis and lateral slip of TP.
- Angle was defined between I. metatarsal bone axis and lateral border of FHB tendon.

### 2.2 Statistical Analysis

Normality was assessed with the Shapiro–Wilk test. Non‐parametric comparisons used the Mann–Whitney *U* and Kruskal–Wallis tests. Correlations were evaluated by Spearman's rho; categorical data by chi‐square or Fisher's exact tests. Analyses were performed in Statistica v13.3.1 (TIBCO Software Inc., USA); *p* < 0.05 was considered significant.

## 3 Results

### 3.1 General Findings

**FIGURE 3:**
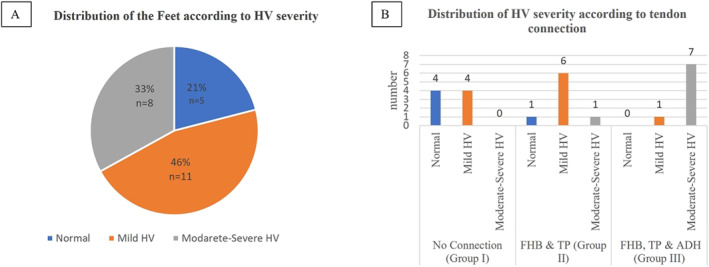
The distribution of the feet according to the HV severity is shown in (A), and the distribution of the HV severity to the tendon connection is shown in (B). ADH: adductor pollicis muscle and FHB: flexor hallucis brevis muscle, HV: hallux valgus, TP: tibialis posterior muscle.

**FIGURE 4:**
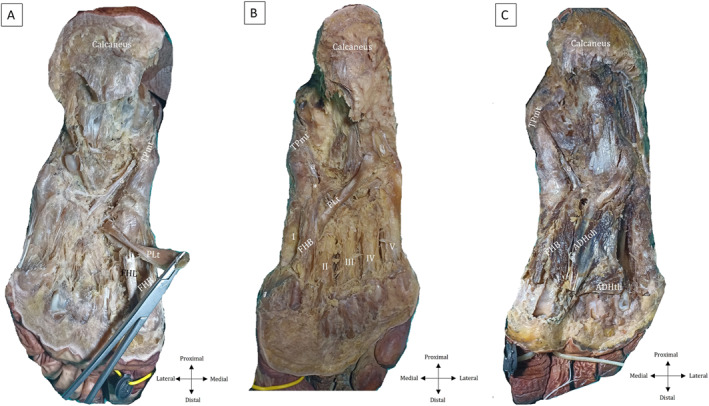
Status of the feet's muscle tendinous connection. (A) Group I ‐ No connection, (B) Group II – Connection between TP & FHB, (C) Group III ‐ Connection between TP, FHB & ADH. *: shows the tendon connection, I‐V: 1‐5 metatarsal bones, a: lateral slip of TPMT, ADHoh: oblique head of adductor pollicis muscle, ADHth: transverse head of adductor pollicis muscle, FHB: flexor hallucis brevis muscle, FHL: flexor hallucis longus tendon (cut), PLt: peroneus longus tendon, TPmt: main tendon of the tibialis posterior muscle.

The distribution of the feet according to the HV was found as normal in 5 cases (3 males and 2 females) (Figures 1B and 3A), mild HV in 11 cases (6 males and 5 females) (Figures 1C and 3A), and moderate/severe HV in 8 cases (5 males and 3 females) (Figures 1D and 3A). In classification according to muscle connection status, It was recorded that there was no muscle connection (Group I, *n* = 8) (Figures 3B and 4A), a single connection between FHB & TP (Group II, *n* = 8) (Figures 3B and 4B), and multiple connections between FHB, TP and ADH (Group III, *n* = 8) (Figures 3B and 4C). No cases were found to be linked solely to TP and ADH. In all cases, TP was linked to FHB and ADH.

### 3.2 Findings About the Relation Between HVA, HV Severity, and Muscle Connection Status

**FIGURE 5:**
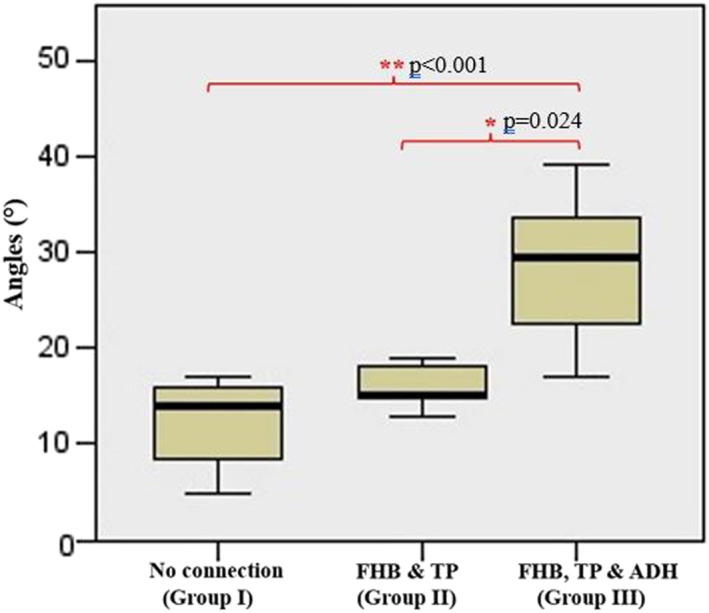
Comparison of HVAs between all groups. ADH: adductor pollicis muscle and FHB: flexor hallucis brevis muscle, TP: tibialis posterior muscle.

There was no relation between sex, side, HV severity and muscle connection status (*p* > 0.05). According to direct measurements, there was no statistical significance between age and HVA (*p* > 0.05). However, there was a strong negative correlation between foot length and HVA (*r* = −0.683, *p* < 0.001). There were statistically significant relationships between muscle connections (*p* < 0.001), the number of connections (*p* < 0.001), and HV severity. Firstly, the feet were classified into two groups with and without muscle connections. The mean HVAs were statistically higher in the group with muscle connection compared to the other group (*p* = 0.005). Additionally, in Group III (FHB, TP & ADH), the mean HVAs were found to be higher and statistically significant compared to Groups I (no connection) and II (FHB & TP) (*p* < 0.001 and *p* = 0.024, Figure 5). However, no statistically significant difference was detected between Group I and II.

### 3.3 Findings About the Muscle Angulation Status

Descriptive statistics of angles regarding all groups were given in Table 1. There was no relation between sex, side, and age regarding muscle angulation status (*p* > 0.05). According to direct measurements, there was no statistical significance and correlation between HVA and muscle angulation status (*p* > 0.05). However, there was a strong positive correlation between cuneometatarsal and TP‐metatarsal angles (*r* = 0.602, *p* = 0.002). It was determined that there was no statistically significant difference in terms of muscle angulation averages in feet with and without muscle connection and between muscle connection groups.

**TABLE 1:** Descriptive statistics of muscle‐tendon angulation status.

Groups	Angles	Mean ± SD	Median [25%–75%]	Min–Max
No connection (Group I)	HV	13.38° ± 4.84	14° [6.75–16.00]	5.00°–17.00°
	CM	24.89° ± 7.49	25° [19.00–31.50]	13.00°–36.00°
	TP‐MT	43.89° ± 11.96	45° [36.50–50.00]	22.00°–65.00°
	TP‐FHB	111.17° ± 14.99	111° [97.25–123.50]	95.00°–131.00°
FHB + TP (Group II)	HV	19.75° ± 11.95	15° [15–18.5]	13.00°–49.00°
	CM	29.00° ± 8.67	28° [23.25–38.5]	16.00°–41.00°
	TP‐MT	49.13° ± 23.17	44.5° [34–62.25]	24.00°–97.00°
	TP‐FHB	99.67° ± 23.64	102.5° [78–120.75]	66.00°–126.00°
FHB + TP & ADH (Group III)	HV	28.38° ± 7.48	29.5° [21.25–34.75]	17.00°–39.00°
	CM	23.50° ± 7.15	22° [17–31.5]	16.00°–34.00°
	TP‐MT	39.75° ± 8.10	40.5° [35.00–45.50]	25.00°–52.00°
	TP‐FHB	108.38° ± 16.32	102.5° [99.25–111.00]	98.00°–147.00°

*Note:* The descriptions of the parameters are given as A & B in Figure 2 respectively.

## 4 Discussion

We found that the TP, especially its tendon extensions, may be related to the formation of HV. Moreover, it is observed that the severity of HV is greater in feet where there is a connection between these tendon extensions and the FHB and ADH. It was recorded that HV was observed whether there was a tendon connection or not. In addition to this information, the angles of the tendon extensions and intrinsic muscles with the os metatarsal I axis were evaluated and it was revealed that the positional angulations of the muscles were not related to HV. Our study strongly supports the literature [5] that there may be many natural or acquired biomechanical abnormalities in feet with HV and that several factors may come together for the occurrence and diagnosis of HV in any individual.

### 4.1 Evaluation of the Tibialis Posterior Tendon Extensions and Connections With Foot Intrinsic Muscles Regarding the Hallux Valgus

In our previous study, we evaluated the positional changes of extensor hallucis longus and brevis in HV conditions in relation to this pathogenesis [20]. We also showed that the smaller tendon size of two intrinsic muscles (one plantar and one dorsal) and one extrinsic muscle in the moderate/severe HV group and the changes in tendons were evident in the cases of high‐severity HV but not in the cases of mild HV [20]. However, there is no clear statement in the literature that the connection of the TP with the ADH or FHB may be involved in the etiology of HV. The widening of the TP tendon toward the ADH is a known variation in the literature. However, the first reference to HV was in the study conducted by Gunal et al. [21] where they described the connection between TP and ADH in all feet of cadavers with HV but reported that they never encountered it in feet without any pathology [21]. Günal et al. [21] suggested that widening caused by TP could be considered a contributing factor in the etiology of HV, along with other factors. Additionally, even suggested that this widening be removed in addition to other operative procedures selected for the surgical treatment of patients with HV [21]. In our study, in support of Chhaya et al. [7] the observation of HV was similar when TP was associated with ADH and FHB. However, we found that HV could also be observed without any muscle connection. In conclusion, we demonstrated that HV can occur in all feet with or without tendon attachment, but HV is more common in cases where the TP is connected to the FHB and ADH, and the frequency of moderate‐to‐severe HV increases in cases where the TP is connected to the ADH. This could be explained by the hypothesis proposed by Chhaya et al. [7] that the main factor in the development of HV is genetic and geometric variations may lead to changes in biomechanics [7].

It was reported that abnormal placement of the TP tendon may be related to HV development, but this relationship does not clearly state whether it is presented as biomechanical or qualitative [24]. In a cadaveric study, the ADH had a consistent fascial sling in the hindfoot and an insertion through the tibial sesamoid [25]. Simulated contraction of this muscle results in flexion and supination of the first metatarsal, elevating the arch and resisting HV. In addition to the role of extrinsics such as the gastrocnemius in driving equinus and the TP tendons in resisting hindfoot deformity, the ABH may contribute as an intrinsic muscle involved in developing HV [25]. TP tendon dysfunction is known to be the most common cause of pes planus [12, 13]. Despite the common belief that pes planus plays an important role in HV, there is convincing pedobarographic and radiographic evidence to the contrary [2, 3, 4, 26]. In addition, it was reported that there was no significant relationship between the co‐observation of HV and several different measurements used to determine pes planus [4]. Here the question goes toward, Is the connection of TP with ADH or FHB located lateral to the first metatarsal? If so, could this extrinsic muscle help pull or pronate the proximal phalanx laterally by contributing to both intrinsic muscles? In our study, these attachments were located laterally in all feet, but we do not have any quantitative information on the biomechanics of the attachments and muscles. Quantitatively, we evaluated the axis of insertion of the tendons into the bone and the angulation of the connection with the axis of the first metatarsal and phalanx. No significant relationship was found. In addition, our study is the first to evaluate the axis of attachment of tendons to the bone and the relationship of the connection with the first metatarsal and phalanx axis.

On the other hand, changes in the morphological properties of the plantar fascia and intrinsic muscles of the foot may be another factor in the emergence of HV [18]. It has been reported that this condition may be related to the morphological properties of the ADH and FHB, such as thickness and cross‐sectional area, and muscle function, such as muscle strength [18]. Reduced stiffness in the intrinsic muscles and the plantar fascia may lead to a reduced capacity to resist external loading, and it has been suggested that this may cause loss of stabilization of the first metatarsophalangeal joint, leading to HV. The ABH and FHB have lower thickness and cross‐sectional area in individuals with HV compared to individuals without HV [16, 17]. It has not been fully explained whether the decrease in AbH and FHB muscle strength causes HV or whether HV causes a decrease in ABH and FHB muscle strength [18]. There is no evidence that the changes in this cause HV, but it may have affected the progression of HV. The connection of the TP with other muscles, which was also included in our study, namely the deviations in the insertion of the TP, may also affect the function of the muscle/s positively or negatively.

Another proposed intrinsic causal factor for HV is the imbalance in muscle strength between the ABH and ADH [15, 27, 28]. In previous electromyographic studies, some investigators reported that the activity of the ABH muscle during abduction of the first MTP joint was significantly reduced in the HV group compared to the activity of the AddH muscle during adduction of the first MTP joint in a control group [15, 27]. Therefore, some authors have referred to strengthening the ABH muscle, which can correct the HV, at least in the early stages [15]. Treatment of the first MTP, HV targets the pulling moment of the big toe and first metatarsal, and both muscles are strengthened [15, 29, 30]. In HV, the misalignment of the first MTP joint directs the net intrinsic muscle moment from plantarflexion to abduction [15]. The ADH counterbalances the ABH in supporting the alignment of the first MTP joint [15, 31]. It has been reported that the oblique head is developmentally large, supports the first ray of movement in weight bearing, and degenerates as the oblique head is not used [32]. Additionally, evolution has reduced the ability of humans to move the big toe with true independence [32]. One conclusion to be drawn from this is that the “activation dominance” of the ADH may not initiate the HV as suggested, and since the oblique head is the only part of the muscle that is placed directly over the hallux, the small and mostly tendinous head exerted by this oblique head can never overpower the opposing ABH [32]. However, we speculate that the connection with the TP may upset this balance by providing extra force to the ADH.

### 4.2 Limitations

Dissected feet were performed with 10% formalin fixation. It has been well described in the literature that fixation may show physical and histological differences in tendon structures [33]. All groups were exposed to the same fixative solution. Therefore, the possible effects of fixation on the shortening and stiffening of the muscles and changes in the joint ligaments were ignored in comparisons.

A second limitation is that although it is generally known that the forefoot geometry can change when weight‐bearing, we had to measure HVA under non‐weight‐bearing foot conditions in this study.

This study was limited to 24 feet, a relatively small sample size. Further studies, the case number can be increased, more effective results can be obtained by including male and female, and even age groups.

## 5 Conclusion

Connections were present in 64% of mild and 100% of moderate‐severe cases; HV occurred in 50% of feet without connections versus 87.5% (100% in the moderate‐severe group) of feet with connections. These findings are indicating that intrinsic muscle attachments to the TP may be associated with the presence and progression of HV. In this case, we speculate that the connection with TP may have disrupted this balance by providing extra force to ADH. We emphasize that in the development and severity of HV, the muscles of the foot, as well as intrinsic muscles, should be addressed. In addition, we believe that testing the hypothesis with biomechanical studies will contribute to conservative or surgical treatment planning and even to determining the treatment according to the individual. Further studies may link cadaveric findings with clinical results of anatomical devices in vivo (e.g. ultrasound or gait simulation). In particular, prior to HV correction surgery, potential muscle connections should be confirmed with ultrasound and MRI. Previously recommended HV correction procedures should be reviewed, with existing connections being severed. Clearly, further clinical studies are still needed to confirm this.

## Author Contributions

**Turan Koç:** conceptualization, data curation, methodology, investigation, formal analysis, writing – original draft, writing – review and editing. **Zeliha Kurtoğlu Olgunus:** conceptualization, methodology, supervision, formal analysis, writing – review and editing. **Fatih Çiçek:** data curation, investigation, writing – review and editing. **Alev Bobuş Örs:** conceptualization, investigation, methodology, supervision, writing – review and editing.

## Ethics Statement

The study has been approved by the Board of Ethics of Mersin University (Approval number: 2021‐96 and 2022‐573).

## Conflicts of Interest

The authors declare no conflicts of interest.

## Data Availability

The data (accessible part) presented in this study is available upon reasonable request to the corresponding author.
